# Baseline levels of dynamic CD4^+^ T cell adhesion to MAdCAM-1 correlate with clinical response to vedolizumab treatment in ulcerative colitis: a cohort study

**DOI:** 10.1186/s12876-020-01253-8

**Published:** 2020-04-15

**Authors:** Clarissa Allner, Michaela Melde, Emily Becker, Friederike Fuchs, Laura Mühl, Entcho Klenske, Lisa Müller, Nadine Morgenstern, Konstantin Fietkau, Simon Hirschmann, Raja Atreya, Imke Atreya, Markus F. Neurath, Sebastian Zundler

**Affiliations:** Department of Medicine 1, University Hospital Erlangen, Friedrich-Alexander-Universität Erlangen-Nürnberg, Kussmaul Campus for Medical Research & Translational Research Center, Ulmenweg 18, 91054 Erlangen, Germany

**Keywords:** Inflammatory bowel diseases, T cells, Vedolizumab, Adhesion, Gut homing

## Abstract

**Background:**

While the number of therapeutic options for treating inflammatory bowel diseases (IBD) is increasing, evidence for rational treatment decisions is scarce in many cases. In particular, appropriate biomarkers to predict the response to the anti-α4β7 integrin antibody vedolizumab are currently lacking.

**Methods:**

We performed a cohort study with 21 patients suffering from ulcerative colitis (UC), in which first-time treatment with vedolizumab was initiated. CD4^+^ T cells were isolated from the peripheral blood and dynamic adhesion to recombinant mucosal vascular addressin cell adhesion molecule (MAdCAM-)1 in vitro as well as the effect of vedolizumab on such adhesion in vitro was determined. The expression of α4β1 integrin on peripheral blood CD4^+^ T cells was quantified by flow cytometry. Electronic patient records were reviewed to determine clinical response to vedolizumab.

**Results:**

Dynamic adhesion of peripheral blood CD4^+^ T cells to MAdCAM-1 and the reduction of adhesion following vedolizumab treatment in vitro were higher and the change in α4β1 expression on CD4^+^ T cells was different in vedolizumab responders and non-responders. Responders could be identified with high specificity and positive-predictive value.

**Conclusions:**

Determining dynamic adhesion of CD4^+^ T cells to MAdCAM-1 and the in vitro response to vedolizumab before treatment initiation or dynamic integrin regulation in the early course of treatment seem to be promising tools to predict the clinical response to vedolizumab therapy. Larger prospective studies are warranted.

## Background

Despite an increasing therapeutic armamentarium for the treatment of inflammatory bowel diseases (IBD), disease activity can still not be sufficiently controlled in a considerable number of patients [1]. Response to the available agents is observed only in portions of patients [2–4] and, additionally, patients may lose response over time [5]. Moreover, there is evidence indicating that the probability of response to a subsequent treatment is lower, if previous therapies have failed [6], and health care systems may be encumbered with costs for ineffective therapies [7].

Thus, treatment selection in individual patients remains an important challenge. Since head-to-head studies and biomarkers for the prediction of response to therapy are largely lacking, objective guidance driving such treatment decisions is low.

The anti-α4β7 antibody vedolizumab is successfully used for the treatment of IBD since 2014 [3, 8] and has been shown to inhibit immune cell homing to the inflamed gut [9, 10] indicating that cell trafficking is a central event in the pathogenesis of IBD [11]. Randomized controlled trials [3, 8], as well as several real-world cohorts [12–14], demonstrated the efficacy and safety of vedolizumab in ulcerative colitis (UC) and Crohn’s disease (CD). Vedolizumab is considered to be rather “slow-acting” [15], which might be explained by its mode of action, not directly targeting intestinal immune cells but only their replenishment by recruitment of cells from the peripheral blood [16]. Therefore, to avoid long periods of ineffective treatment in non-responders, the identification of biomarkers to predict response to vedolizumab therapy is a particularly unmet need. Moreover, since vedolizumab rather acts in the peripheral blood than in intestinal tissue, the drug might provide an especially convenient opportunity for the determination of biomarkers with low invasiveness.

We had previously introduced a dynamic adhesion assay to study the adhesion of immune cells to cell adhesion molecules [17]. In this context, we had reported the anecdotal observation that the extent of dynamic adhesion of peripheral blood CD4^+^ T cells from IBD patients to the α4β7 ligand mucosal vascular addressin cell adhesion molecule (MAdCAM-)1 in this assay before initiation of vedolizumab treatment seemed to correlate with subsequent clinical response to therapy.

Here, we conducted a retrospective cohort study in UC patients treated with vedolizumab to further investigate this hypothesis. We show that dynamic adhesion to MAdCAM-1 is higher in responders than in non-responders and that vedolizumab treatment in vitro leads to a stronger reduction of adhesion in responders compared with non-responders. High levels of dynamic adhesion had a high specificity and positive predictive value for the response to treatment.

## Methods

### Patients with IBD

After informed written consent, we collected peripheral blood from adult patients with an established diagnosis of UC (*n* = 23) directly before the initiation of first-time vedolizumab treatment at the IBD Outpatient Department of the Medical Clinic 1 of the University Hospital Erlangen. From some patients, additional blood samples were collected after 6 weeks of treatment. The procedures were approved by the institution’s ethics committee (Ethics Committee of the Friedrich-Alexander-University Erlangen-Nuremberg). Vedolizumab therapy was conducted according to standard clinical protocols.

Table 1 summarizes the donors’ baseline characteristics.

**Table 1:** Baseline characteristics of included patients

	Ulcerative Colitis
**Number of patients**	21
**Age (mean, range)**	36.8 (19–70)
**Female [%]**	48
**Smoking status [%]**	
Never	95
Current	5
**Mayo clinical score (mean, range)**	4.1 (1–7)
**Adjunctive therapy [%]**	
Steroids	42.9
Immunosuppressants	14.3
**Previous exposure to anti-TNF [%]**	76
**Previous surgery [%]**	0
**Localization [%]**	E1: 19
	E2: 24
	E3: 57

### Cell isolation and in vitro treatment

Peripheral blood mononuclear cells (PBMCs) were obtained by standard density gradient centrifugation with Lymphocyte Separation Medium (Anprotec). Subsequently, CD4^+^ T cells were isolated from PBMCs with immunomagnetic beads (Miltenyi Biotec) according to the manufacturer’s instructions. CD4^+^ T cells were fluorescently labeled with carboxyfluorescein succinimidyl ester (CFSE; Life Technologies). Labeled cells were resuspended at 1.5 million cells/mL in RPMI 1640 medium (Thermo Fisher) with 1% penicillin/streptomycin (Biochrom) and 10% FCS (Pan Biotech) and incubated with or without the anti-α4β7 integrin antibody vedolizumab (10 μg/mL, Takeda) in vitro. Finally, cells were harvested and resuspended in adhesion buffer (pH 7.4; 150 mM NaCl, 10 mM HEPES, 1 mM CaCl2, 1 mM MgCl2, 1 mM MnCl2) at a concentration of 1.5 million cells/mL for subsequent use in dynamic adhesion assays.

### Dynamic live cell in vitro adhesion assays

Dynamic live cell adhesion was analysed as determined previously [17, 18]. In brief, the inside of miniature borosilicate capillaries (Vitrocom) was coated with Fc chimera of rhMAdCAM-1 (R&D Systems) at a concentration of 5 μg/mL in 150 mM NaCl with 10 mM HEPES for 1 h at 37 °C. Next, the coating solution was carefully removed and unspecific binding sites were blocked with 5% bovine serum albumin (BSA) in phosphate buffered saline (PBS) for another hour at 37 °C. Capillaries were connected to plastic tubing, which was inserted in an adjustable flow rate peristaltic pump (Baoding Shenchen Precision Pump Company).

Perfusion speed was set to 10 μL/min and cells prepared as mentioned above were perfused through the capillaries. Time-lapse confocal microscopy was used to quantify dynamic adhesion to MAdCAM-1 over a 3 min period. Briefly, overlay of three sequential images from the beginning and the end of the sequence in ImageJ (NIH) allowed quantification of adherent cells before and after the adhesion period. The difference was calculated to obtain dynamic adhesion during these 3 mins.

### Outcomes

**Fig. 1:**
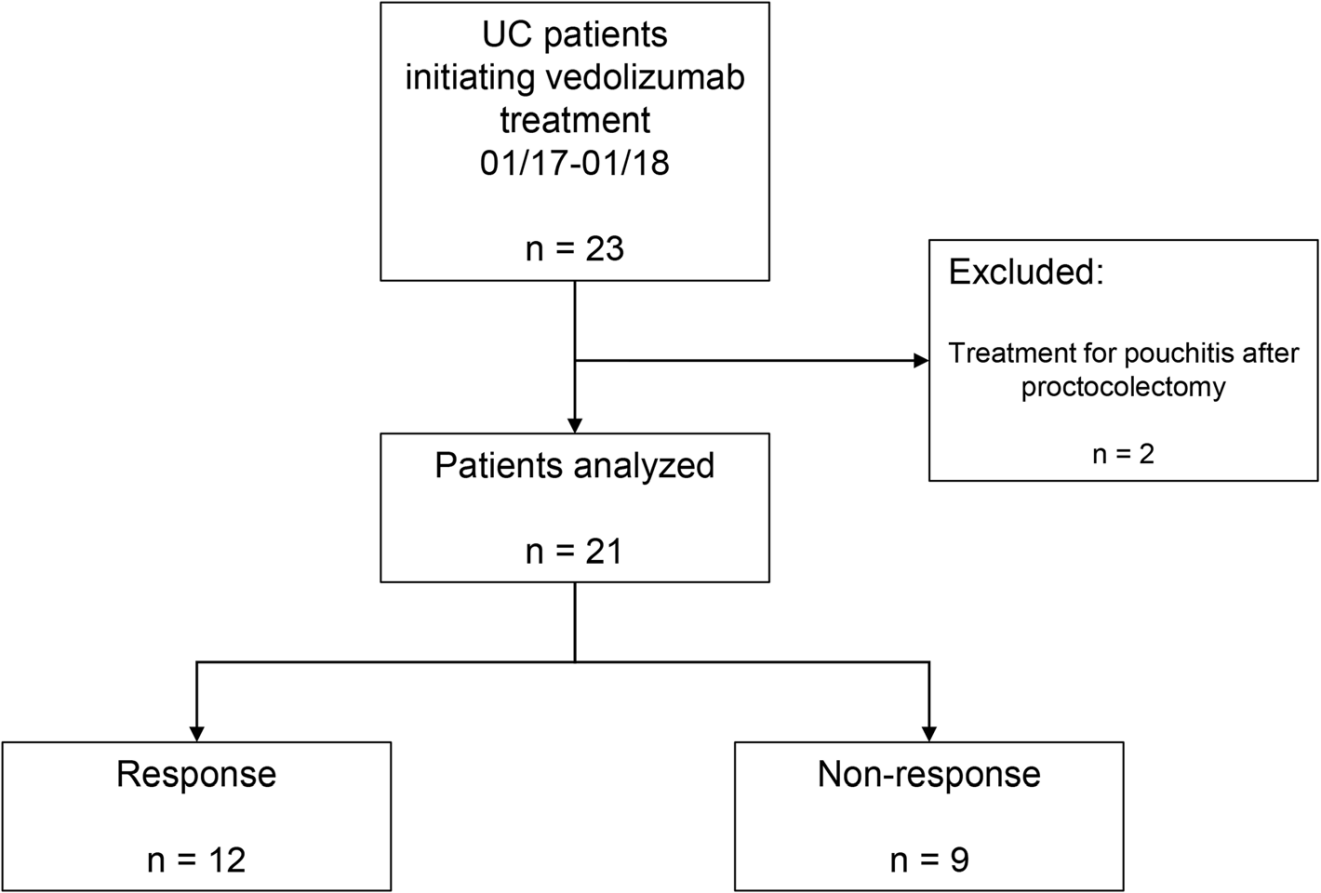
Patient inclusion chart and clinical response

Electronic patient records were retrospectively reviewed to determine clinical response to vedolizumab treatment after 15 weeks (mean 14.7 weeks +/− 0.2 weeks SEM) of therapy. The Mayo Clinical Score (MCS, i.e. non-invasive 9 point-score [19]) was used to determine clinical disease activity. Two patients that had previously undergone proctocolectomy and received vedolizumab for pouchitis were excluded from the intention-to-treat analysis (Fig. 1).

Clinical response was defined as a reduction of MCS >/= 2 or, alternatively, steroid-free remission (MCS <  2) in patients who initiated treatment in steroid-dependent clinical remission.

### Flow Cytometry

For the analysis of α4β1 expression on CD4^+^ T cells from the peripheral blood, PBMCs were isolated, stained with antibodies against CD4 (VioBlue, VIT4; Miltenyi Biotec), α4 integrin (FITC, MZ18-24A9; Miltenyi Biotec), β1 integrin (AF647, TS2/16; Biolegend), and fixed with the FoxP3/Transcription Factor Staining Buffer Set (eBioscience). Flow cytometry was performed on LSR Fortessa (BD) and MACSQuant (Miltenyi Biotec) instruments.

The difference in α4β1 expression on CD4^+^ T cells between week 0 and week 6 was calculated. Amending the data available from a previously published cohort [20], α4β1 expression differences in responders and non-responders were analysed.

In a separate analysis, we stained PBMCs with antibodies against CD4, α4 integrin and β7 integrin (PerCP-Cy5.5, FIB27; Biolegend). Additionally, vedolizumab was labeled with AF647 (AF647 labeling kit; ThermoFisher) and used to stain these PBMCs at a concentration of 10 μg/ml. The rate of vedolizumab^+^ cells was determined after gating on CD4^+^α4^+^β7^+^ cells.

### Statistics

Graph Pad Prims (Graph Pad Software) was used to perform statistical comparisons. Adhesion and expression levels were compared with Mann-Whitney test. Bar charts display mean values with SEM. Cut-offs were determined by receiver operator characteristics (ROC) analysis. Significance levels are indicated by asterisks (* *p* < 0.05, ** *p* < 0.01, *** *p* < 0.001).

## Results

### Higher dynamic adhesion of CD4^+^ T cells from UC patients to MAdCAM-1 in responders to vedolizumab therapy compared with non-responders

From the 21 vedolizumab-treated patients with UC that underwent analysis, 12 (57.1%) had a clinical response, while 9 (42.9%) were non-responders (Fig. 1).

In the dynamic adhesion assays performed in these patients, almost no adhesion to negative control capillaries was observed (Suppl. Fig. 1). Moreover, we performed flow cytometric analyses to demonstrate that the vedolizumab concentration used indeed leads to substantial binding to α4β7 integrin-expressing CD4^+^ T cells (Suppl. Fig. 2).

**Fig. 2:**
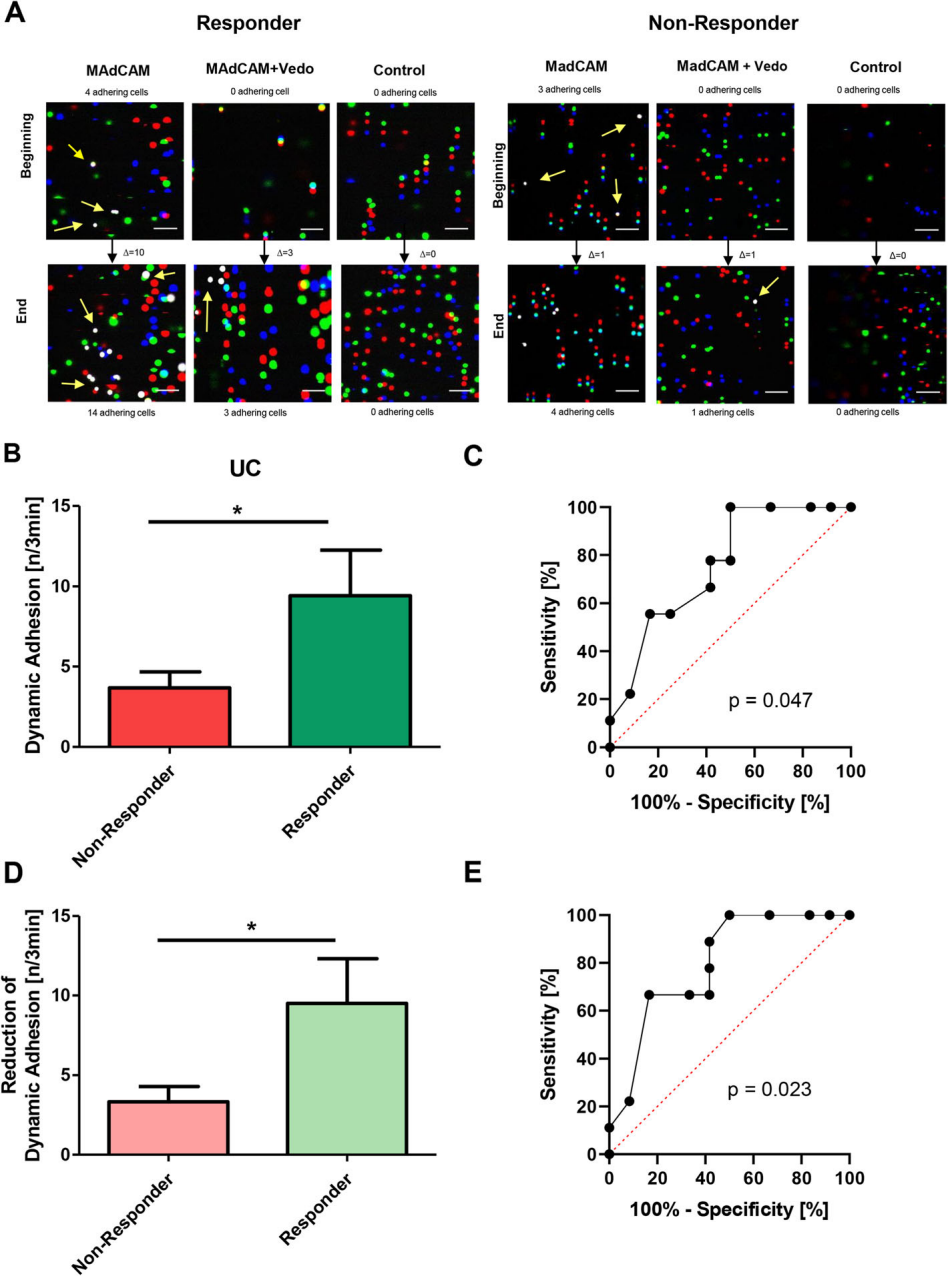
Dynamic adhesion of CD4^+^ T cells to MAdCAM-1 in the cohort of 21 UC patients. **a** Representative dynamic adhesion assays from a responder (left) and a non-responder (right) to vedolizumab treatment. Overlay images from the beginning and the end of the collected time-lapse sequences with enumeration of adhering cells (white, some of them highlighted with yellow arrows) are shown. Scale bar – 50 μm. **b** Dynamic adhesion of CD4^+^ T cells to MAdCAM-1 in responders (*n* = 12) vs. non-responders (*n* = 9). Statistical comparison with two-tailed Mann-Whitney test. **c** Receiver-operator characteristic (ROC) analysis of the dynamic adhesion of CD4^+^ T cells to MAdCAM-1 in responders vs. non-responders. The *p*-value is indicated. **d** Reduction of dynamic adhesion of CD4^+^ T cells to MAdCAM-1 after vedolizumab treatment in vitro in responders (*n* = 12) vs. non-responders (*n* = 9). Statistical comparison with two-tailed Mann-Whitney test. **e** Receiver-operator characteristic (ROC) analysis of the reduction of dynamic adhesion of CD4^+^ T cells to MAdCAM-1 after vedolizumab treatment in vitro in responders vs. non-responders. The *p*-value is indicated

In patients with a response, we observed high numbers of CD4^+^ T cells adhering to MAdCAM-1 in the dynamic adhesion assays, whereas adhesion of CD4^+^ T cells from patients classified as non-responders to MAdCAM-1 was substantially lower (Fig. 2a, b). Therefore, we performed a ROC analysis that revealed an area under the curve (AUC) of 0.76 (95% confidence interval (CI) 0.55–0.97; Fig. 2c). A dynamic adhesion of 8.5 cells/3 min was identified as the best cut-off value, which was associated with a specificity of 100% and a sensitivity of 50% for the identification of response to vedolizumab treatment. The positive and negative predictive values were 100 and 60%, respectively (Table 2A). Additionally, a cut-off value of 2.5 cells/3 min was able to identify non-responders with 83.3% specificity and 55.6% sensitivity. The positive and the negative predictive values were 71.4% (Table 2B).

**Table 2:** Contingency tables and cut-offs

**A**			
	**Response**	**Non-Response**	
Dynamic adhesion > 8.5 cells/3 min	6	0	PPV: 100%
Dynamic adhesion < 8.5 cells/3 min	6	9	NPV: 60%
	Sensitivity: 50%	Specificity: 100%	
**B**			
	**Non-Response**	**Response**	
Dynamic adhesion < 2.5 cells/3 min	5	2	PPV: 71.4%
Dynamic adhesion > 2.5 cells/3 min	4	10	NPV: 71.4%
	Sensitivity: 55.6%	Specificity: 83.3%	
**C**			
	**Response**	**Non-Response**	
Reduction of adhesion > 8.5 cells/3 min	6	0	PPV: 100%
Reduction of adhesion < 8.5 cells/3 min	6	9	NPV: 60%
	Sensitivity: 50%	Specificity: 100%	
**D**			
	**Non-Response**	**Response**	
Reduction of adhesion < 2.5 cells/3 min	6	2	PPV: 75%
Reduction of adhesion > 2.5 cells/3 min	3	10	NPV: 76.9%
	Sensitivity: 66.7%	Specificity: 83.3%	
**E**			
	**Response**	**Non-Response**	
Dynamic adhesion > 8.5 cells/3 min	6	0	PPV: 100%
Reduction of adhesion > 2.5 cells/3 min			
Dynamic adhesion < 8.5 cells/3 min	4	3	
Reduction of adhesion > 2.5 cells/3 min			
Dynamic adhesion < 8.5 cells/3 min	2	6	NPV: 75%
Reduction of adhesion < 2.5 cells/3 min			

In a separate analysis, we evaluated whether the extent of reduction of dynamic adhesion of CD4^+^ T cells to MAdCAM-1 following in vitro treatment with vedolizumab also correlated with the outcome of therapy. Indeed, the reduction of dynamic adhesion was more pronounced in responders compared with non-responders (Fig. 2d). In ROC analysis, the AUC was 0.80 (95% CI 0.60–0.99; Fig. 2e). Using a cut-off value of a reduction of adhesion of 8.5 cells/3 min, responders to vedolizumab treatment could be identified with 100% specificity and 50% sensitivity. Positive and negative predictive values were 100 and 60%, respectively (Table 2B). Moreover, a cut-off value of a reduction of adhesion of 2.5 cells/3 min distinguished between non-responders and responders with 83.3% specificity and 66.7% sensitivity. The positive predictive value for non-response was 75% and the negative predictive value 76.9% (Table 2D).

Together, high absolute adhesion to MAdCAM-1 seemed particularly suitable to predict response, while low reduction of adhesion was more feasible to predict non-response. Consistently, when combining both parameters, we found that 100% of those patients with an adhesion above 8.5 cells/3 min and a reduction of adhesion induced by vedolizumab treatment in vitro of more than 2.5 cells/3 min had a clinical response, whereas 75% or the patients with an adhesion below 8.5 cells/3 min and a reduction below 2.5 cells/3 min had a non-response. Among the seven patients with adhesion below 8.5 cells/3 min and reduction of adhesion above 2.5 cells/3 min, 57.1% were responders (Table 2E).

### Dynamic changes in α4β1 expression early during vedolizumab treatment correlate with response to therapy

While these data suggested that the function of α4β7 in vitro correlates with clinical response to vedolizumab therapy, we had previously shown that other homing pathways may bypass α4β7 blockade and may in that way modulate clinical effects of vedolizumab. In particular, we have shown that α4β7 blockade may be bypassed by homing of α4β7- and α4β1-expressing T cells via α4β1 in vivo [21]. Moreover, we had previously also suggested that clinical response to vedolizumab treatment at week 16 can be predicted by dynamic changes in α4β1 expression on CD4^+^ T cells from week 0 to week 6 [20], which might also be in line with circumvention of α4β7 blockade by α4β1 as an alternative homing pathway. Therefore, we also analyzed the regulation of α4β1 expression in several additional patients of the present cohort and now report the results of the extended cohort including a total of 26 UC patients.

**Fig. 3:**
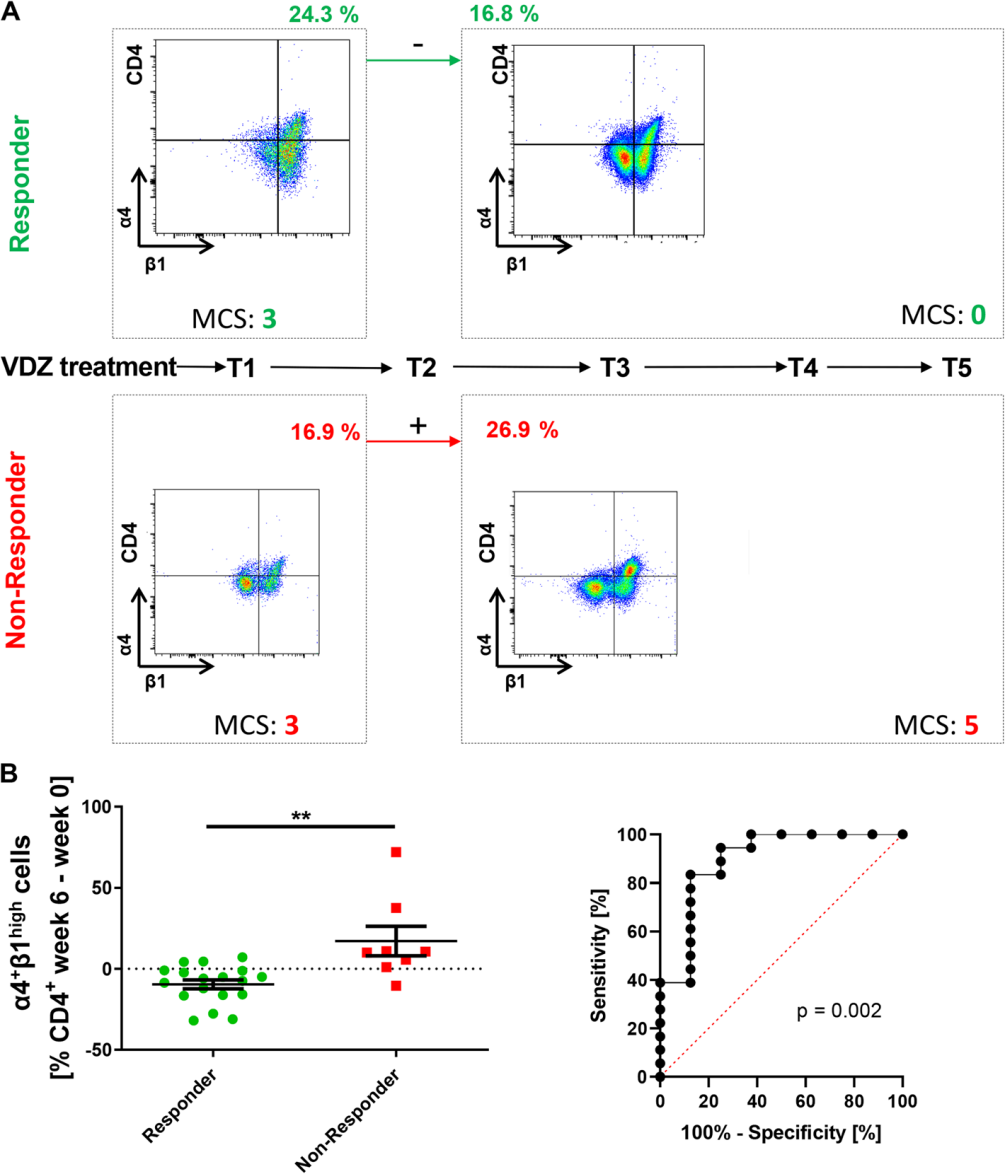
Dynamic expression of α4β1 integrin on CD4^+^ T cells in the peripheral blood of patients with UC treated with vedolizumab. **a** Timeline of vedolizumab treatment (T1–5) with representative flow cytometry and clinical data. Flow cytometry of integrin expression on CD4^+^ T-cells in the peripheral blood was performed before T1 and T3. Clinical response was determined at T5. Representative flow cytometric and clinical data from a responder to vedolizumab (upper panels) and a non-responder to vedolizumab (lower panels) are shown. **b** Left panel: Difference in the expression of α4β1 integrin on CD4^+^ T cells in the peripheral blood between week 0 (T0) and week 6 (T3) of vedolizumab treatment as determined by flow cytometry (*n* = 26; 18 responders, 8 non-responders). Statistical comparison with two-tailed Mann-Whitney test. Right panel: Receiver-operator-characteristic (ROC) analysis for prediction of clinical response to vedolizumab treatment by changes in α4β1 expression between week 0 and week 6. Significance level (left) and *p*-value (right) are indicated. MCS – Mayo clinical subscore

In the previously reported cohort, all responders to vedolizumab treatment had decreasing α4β1 levels on CD4^+^ T cells and all non-responders had increasing α4β1 levels on CD4^+^ T cells. Although we now observed some exceptions from this distribution, there was still a significant and substantial difference between the development of α4β1 expression from week 0 to week 6 (Fig. 3a, b). In the ROC analysis the AUC was 0.90 (95% CI 0.75 to 1.00; Fig. 3b). For the determination of clinical response to vedolizumab therapy, the best sensitivity of 83.3% and specificity of 87.5% was achieved by a cut-off of 0.05% expression change of α4β1 on CD4^+^ T cells in week 6 compared with week 0. This was associated with a positive predictive value of 93.8% and a negative predictive value of 70.0%.

## Discussion

Treatment decisions are a constant challenge in IBD. While more and more agents are available to treat UC and CD, there is no clear sequence of preferred treatments [22] and, thus, the choice of treatment often remains an individual consideration. In some cases, the presence of complications like fistulae [23] or associated conditions like arthritis [24] may help to decide, but in most cases, not evidence-based aspects like patient preferences may be even more important. Apart from one very recent example comparing the efficacy of vedolizumab and adalimumab for achieving clinical remission at week 52 in UC [25], prospective head-to-head studies comparing biological treatments for IBD are lacking. Since it is not likely that appropriate additional randomized head-to-head studies will be performed in the near future to sufficiently guide treatment decisions in particular treatment situations, the identification of biomarkers predicting the individual response or non-response of patients to certain agents is an alternative strategy to make IBD therapy more efficient.

The feasibility of this concept has been previously demonstrated in studies showing that the quantification of target cells for the anti-TNF-α antibody adalimumab during confocal endomicroscopy and the baseline expression of αE integrin predicted subsequent response to adalimumab and the anti-β7 antibody etrolizumab, respectively [26, 27]. With regard to vedolizumab, a broad number of observations has been made: While gene expression profiling identified a considerable number of genes that were significantly differentially expressed following vedolizumab therapy, none of them was able to predict treatment outcomes [28]. However, another study reported that response to vedolizumab was associated with pre-therapeutic composition of the intestinal microbiome [29]. Moreover, we had suggested that dynamic changes in the expression of α4β1 on peripheral blood CD4^+^ T cells in the early course of vedolizumab treatment and the baseline dynamic adhesion of CD4^+^ T cells to MAdCAM-1 in vitro might predict subsequent therapeutic response [17, 20].

Here, we further investigated these hypotheses. Using a dynamic adhesion assay suitable to quantify the adhesion of CD4^+^ T cells to MAdCAM-1 under physiological shear stress [18], we show that in our cohort of 21 UC patients, more T cells from responders than from non-responders adhered to MAdCAM-1 prior to initiation of vedolizumab therapy. Consistently, in vitro treatment with vedolizumab reduced adhesion in responders stronger than in non-responders. AUCs determined with ROC analyses were around 0.8. This corresponds to a fair to good test accuracy [30] and is comparable with the accuracy of fecal calprotectin quantification for the prediction of endoscopic remission in UC [31]. Contingency table analyses showed that the ideal cut-off values determined in ROC analyses for the identification of responders either based on adhesion to MAdCAM-1 or on the reduction of adhesion after in vitro treatment with vedolizumab had high specificity, but only moderate sensitivity due to a number of false-negative results. However, this also mirrored in high positive predictive rates. Consistently, lower cut-off values were better suited to identify non-responders. When combining the information on adhesion to MAdCAM-1 and on the reduction of adhesion by vedolizumab, it turned out that high adhesion and high reduction were strongly associated with response to treatment, while low adhesion and low reduction were clearly associated with non-response. On the contrary, medium levels of adhesion and reduction were not conclusive in this regard.

Taken together, our data suggest that determining the baseline adhesion of CD4^+^ T cells to MAdCAM-1 and their response to vedolizumab in vitro with the proposed assay may be suitable as a functional biomarker and help to support treatment decisions in most patients. It is important to mention, that – based on previous observations [17] – differential levels of CD4^+^ T cell adhesion to MAdCAM-1 are most likely not a result of differential α4β7 integrin expression, but rather differential intrinsic functionality of this pathways in responders vs. non-responders.

We also continued to explore the suitability of changes in α4β1 integrin expression on CD4^+^ T cells early during vedolizumab treatment as a marker of subsequent treatment success and report the extension of a previous cohort [20] in this study. In the expanded cohort of 26 UC patients, on average, CD4^+^ α4β1 integrin levels declined in patients with a clinical response at week 16 within the first 6 weeks of therapy, while it increased in non-responders. Again, specificity and positive predictive value for the assessment of clinical response were high. The mechanistic explanation for this finding might be that α4β1 has been shown to represent an alternative pathway for lymphocyte gut homing, when α4β7 is blocked [21]. Thus, the determination of α4β1 integrin expression might present a promising way to predict the response to vedolizumab as a “slow-acting” drug early in the course of treatment.

It has to be underscored that several limitations have to be taken into account, when interpreting these data. For one aspect, they are based on an only small cohort from one single center. Moreover, clinical parameters were determined retrospectively. Thus, our study needs to be understood as a pilot study and the data cannot easily be generalized and conclusions must be drawn carefully.

Yet, these results show the promising potential of these techniques and provide a clear rationale for subsequent prospective studies with higher sample size, which are currently under way.

## Conclusion

Analysis of the adhesion of CD4^+^ T cells to MAdCAM-1 on a functional level with dynamic adhesion assays in IBD patients prior to the start of treatment appears to be a promising tool for the identification of patients, which are likely to respond to therapy with vedolizumab. Similarly, changes in α4β1 integrin expression after initiation of treatment might be an early indicator of clinical success in vedolizumab-treated patients.

Provided that these findings can be confirmed and validated in larger and prospective studies, our observations might lay the basis for the prediction of success of vedolizumab treatment with the help of peripheral blood samples.

## Supplementary information

**Additional file 1: Figure S1.** Comparison of dynamic adhesion of CD4^+^ T cells from the 21 UC patients of the cohort to MAdCAM-1 and to uncoated negative control capillaries. Comparison with two-tailed Mann-Whitney test. The significance level is indicated.

**Additional file 2: Figure S2.** Flow cytometry of vedolizumab binding to α4β7 integrin-expressing CD4+ T cells. Left: Representative histogram showing the staining intensity for Alexa Fluor 647 (AF647) with (red) or withouth (blue) treatment with 10 μg/mL AF647-labeled vedolizumab. Right: Quantification (*n* = 4). Comparison with paired student’s t-test. The significance level is indicated.

## Data Availability

The datasets generated and analyzed during the current study are available from the corresponding author on reasonable request.

## Abbreviations

AUC: Area under the curve
BSA: Bovine serum albumin
CD: Crohn’s disease
HBI: Harvey-Bradshaw index
IBD: Inflammatory bowel disease
MAdCAM-1: Mucosal addressin vascular cell adhesion molecule 1
MCS: Mayo Clinical Score
PBMCs: Peripheral blood mononuclear cells
rh: Recombinant human
ROC: Receiver operator characteristics
SEM: Standard error of the mean
UC: Ulcerative colitis
