# Supplementary figures and images for: Baseline levels of dynamic CD4+ T cell adhesion to MAdCAM-1 correlate with clinical response to vedolizumab treatment in ulcerative colitis: a cohort study
Source: BMC Gastroenterol. 2020 Apr 15;20:103. doi: 10.1186/s12876-020-01253-8 (PMC7158080; doi:10.1186/s12876-020-01253-8)

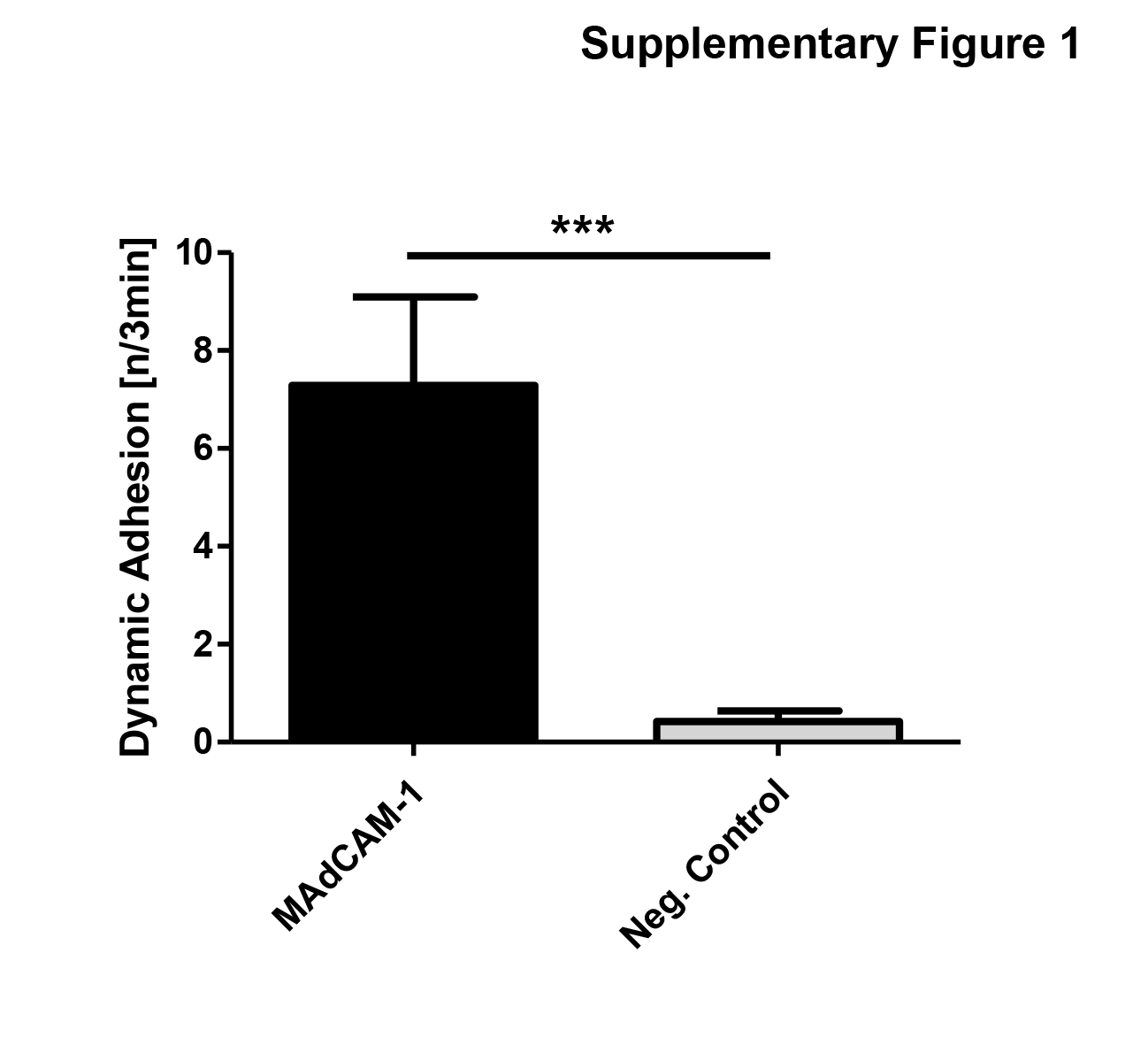

Supplement: Supplementary file 1 — Additional file 1: Figure S1. Comparison of dynamic adhesion of CD4+ T cells from the 21 UC patients of the cohort to MAdCAM-1 and to uncoated negative control capillaries. Comparison with two-tailed Mann-Whitney test. The significance level is indicated. [file 12876_2020_1253_MOESM1_ESM.tif]

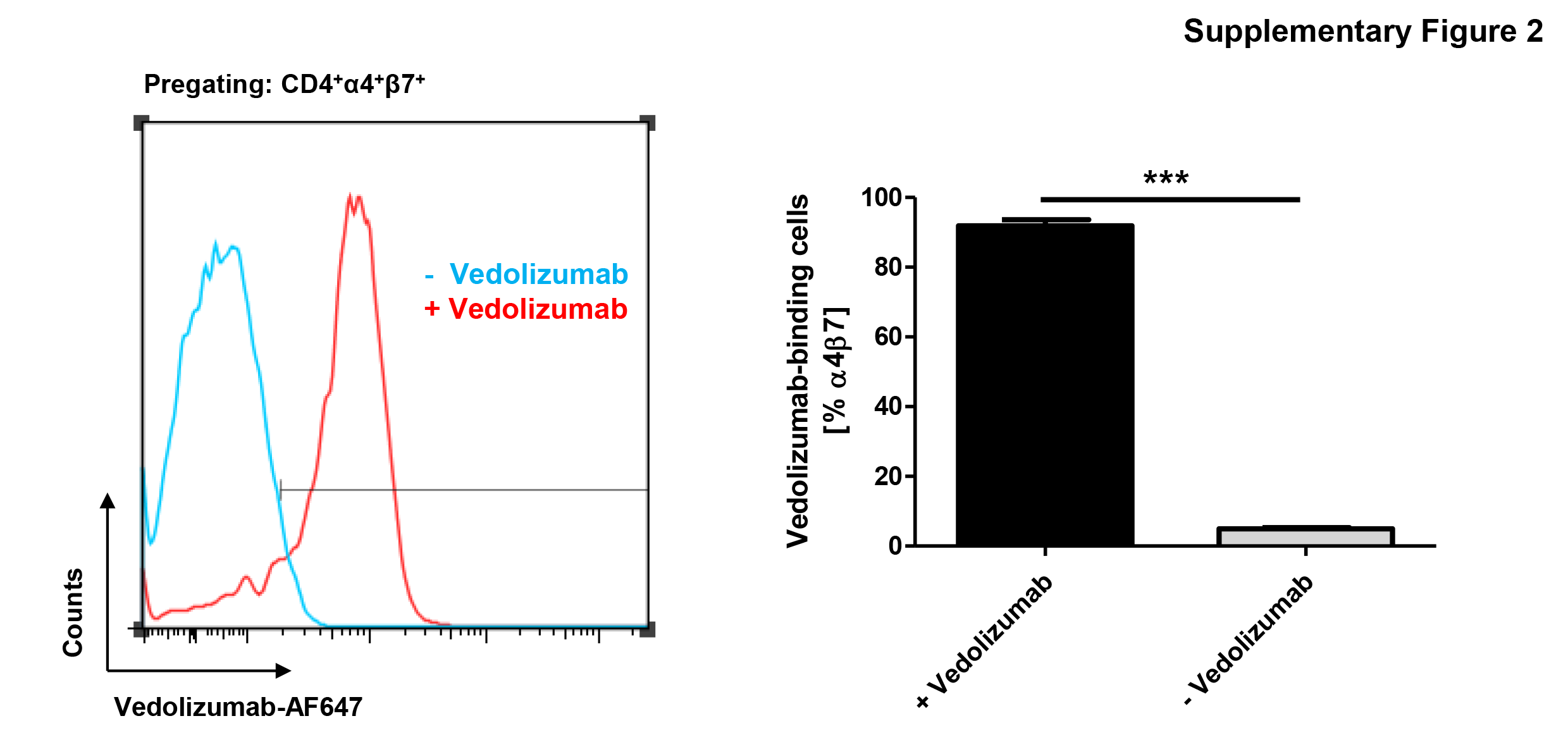

Supplement: Supplementary file 2 — Additional file 2: Figure S2. Flow cytometry of vedolizumab binding to α4β7 integrin-expressing CD4+ T cells. Left: Representative histogram showing the staining intensity for Alexa Fluor 647 (AF647) with (red) or withouth (blue) treatment with 10 μg/mL AF647-labeled vedolizumab. Right: Quantification (n = 4). Comparison with paired student’s t-test. The significance level is indicated. [file 12876_2020_1253_MOESM2_ESM.tif]
